# Evaluation of groundwater quality in the bouanane plain using the groundwater pollution index, nitrate pollution index, and microbiological indicators

**DOI:** 10.1038/s41598-026-44619-w

**Published:** 2026-03-17

**Authors:** Asmae Nouayti, Ali El Mansour, Hamid Nouayti, Abdellali Abdaoui, Radoine Nouayti, Abdelmonaim El mimouni, Mourad Arabi, Saida Ait Boughrous, Hasan Ouakhir, Omar Noman, Abdelaaty A. Shahat, Ali Ait Boughrous, Nadia Lahrach

**Affiliations:** 1 Ethnopharmacology and Pharmacognosy, Faculty of Sciences and Techniques Errachidia, Moulay Ismail University of Meknes, BP 509, Boutalamine, Errachidia Morocco; 2 Laboratoires TBC, Laboratory of Pharmacology, Pharmacokinetics, and Clinical Pharmacy, Faculty of Pharmaceuticaland Biological Sciences, University of Lille, 3, rue du Professeur Laguesse, B.P. 83, F-59000 Lille, France; 3 Bio-resources, Environment and Health, Faculty of Science and Technology of Errachidia, Moulay Ismail University of Meknes, Marjane 2, BP: 298, Meknes, 50050 Morocco; 4 Department of Bioresources, Biotechnologies, Ethnopharmacology, and Health, Faculty of Sciences Oujda, Mohammed First University, Oujda, Morocco; 5 Laboratory of Engineering Sciences and Applications, National School of Applied Sciences, Abdelmalek Essaadi University, Al-Hoceima, 32003 Morocco; 6 Sultan Moulay Slimane University of Beni Mellal, Multidisciplinary Research and Innovation Laboratory (LMRI)/Natural Resource Engineering and Environmental Impacts Team (IRNIE), Polydisciplinary Faculty of Khouribga, BP 145, Khouribga, Morocco; 7 High Institute of Nursing and Health Technology Professions, Demography, Environment, Beni Mellal, 23000 Morocco; 8 Department of Pharmacognosy, College of Pharmacy, King Saud University, P.O. Box 2457, Riyadh, 11451 Saudi Arabia

**Keywords:** Groundwater, Physico-chemical analysis, Bacteriology, NPI, PIG, HHRA, Chemistry, Environmental sciences, Hydrology, Water resources

## Abstract

Groundwater provides a vital component of water supplies in semi-arid environments, wherein its quality directly influences ecosystem stability and human well-being. This investigation presents the first complete evaluation of groundwater quality in the Bouanane basin by implementing an innovative PCA–GIS framework combined with established hydrochemical indices, thereby enhancing the discrimination of pollution sources beyond what conventional methods typically allow. Nine groundwater samples collected in April 2024 were analysed for major ions and microbiological indicators. Water quality was subsequently evaluated using (PIG), (NPI), and a USEPA based Human Health Risk Assessment. PIG values ranged from 1.12 to 3.03 demonstrating that 44% of samples come inside the very highly polluted classification, primarily due to geogenic mineralization associated with carbonate and evaporitic formations. Conversely, NPI values (− 0.98 to − 0.25) indicate negligible nitrate contamination and minimal human influence. Health risk indices for both children and adults remained less than 1, suggesting no significant risk to public health. Although most samples complied with World Health Organization WHO (World Health Statistics, Monitoring Health for the SDGs, Sustainable Development Goals, 2017) drinking water guidelines, Staphylococcus aureus was detected at 22% of sampling locations, underscoring the need for periodic sanitary monitoring. Overall, the findings demonstrate that groundwater chemistry within the basin is predominantly shaped by natural geochemical processes. Furthermore, the integrated PCA–GIS framework proved to be a robust and efficient tool for groundwater quality diagnosis. This pioneering investigation establishes an essential scientific baseline for the Bouanane basin and provides a foundational reference for evidence-based water resource management amid rising climatic and anthropogenic pressures.

## Introduction

Freshwater has historically been fundamental to human advancement. From the earliest settlements established along rivers and springs to modern urban societies, freshwater continues to underpin poverty alleviation, food security, public health, and sustainable socio-economic progress^2,3^. Within global freshwater reserves, groundwater represents the second largest storage component after glaciers and was important for sustaining anthropic and economic activities^4–6^. Around 2.5 billion humans rely predominantly on groundwater for domestic water supply, underscoring its central role in drinking water provision, agriculture, and industry^7^.

Despite its importance, groundwater is increasingly threatened by rapid depletion and declining quality^8–11^. Population expansion, agricultural intensification, industrial growth, and climate related disturbances are amplifying pressures on aquifers, especially in arid and semi-arid regions where ensuring sustainable groundwater management remains highly complex^12–14^.

The Bouanane basin, situated in the Guir watershed of southeastern Morocco, is a semi-arid area hosting significant groundwater resources. Increasing reliance on groundwater for domestic consumption and irrigation has heightened aquifer vulnerability, as reflected by falling piezometric levels, enhanced salinity, and overall degradation of water quality. In some sectors, contamination is directly linked to irrigation return flows, fertilizer application, and the discharge of untreated wastewater.

Previous research undertaken in neighboring watersheds such as Ghris, Oued Guir, and Haut Ziz has primarily focused on physico-chemical or microbiological attributes of groundwater^15,16^. These investigations consistently reported elevated salt and nitrate concentrations, often exceeding drinking water guidelines and raising concerns about long term groundwater sustainability. Nitrate contamination is of particular concern due to its well documented adverse health impacts^17–20^, stressing the critical need for enhanced surveillance and holistic assessment methodologies.

Despite their relevance, existing studies remain methodologically conventional and rely mainly on basic physico-chemical analyses without incorporating advanced pollution indices or quantitative health risk models. No investigation to date has combined physico-chemical and microbiological data with the Nitrate Pollution Index (NPI), the Pollution Index of Groundwater (PIG), and a USEPA based Human Health Risk Assessment (HHRA) within the Bouanane watershed. This methodological gap limits the capacity to trace pollution sources, quantify associated health risks, and design effective groundwater management strategies.

Given the public health implications of nitrate pollution particularly in intensively cultivated semi-arid regions with inadequate sanitation understanding nitrate dynamics in groundwater is essential. Excess nitrate in water consumed by humans is connected with serious health issues, including baby methemoglobinemia, increased cancer risk, and congenital anomalies^17,21,22^. In this framework, the current research employs^21^ guidelines for assess the acceptability of water for domestic usage. Groundwater contamination is further assessed using the PIG, widely acknowledged as a strong measure of aquifer susceptibility^23–26^.

Longterm non carcinogenic health risks associated with nitrate ingestion and dermal exposure are quantified for different age groups using the USEPA based HHRA model^27–30^. To the greatest extent of our understanding, this is the initially thorough research in southeastern Morocco to integrate NPI, PIG, microbiological assessment, and USEPA based HHRA into a unified groundwater quality evaluation framework^22,31^.

The proposed multidisciplinary methodology also incorporates GIS to spatially resolve groundwater quality patterns and delineate zones where nitrate levels may pose health risks. The joint use of PIG and NPI provides complementary benefits: PIG offers a structured classification of pollution severity, whereas NPI specifically targets nitrate a contaminant of major public health relevance. This dual index approach yields a more reliable diagnosis compared with conventional assessments that often overlook regulatory compliance and explicit health risk considerations. International evidence highlights the robustness and policy relevance of such integrative approaches^2,12,26,29,32–41^. Overall, this research aims (i) to assess the physicochemical and bacteriological quality of groundwater in the Bouanane Basin, (ii) identify the dominant geochemical processes shaping groundwater composition, and (iii) evaluate potential humaine health risks with its use. The research also introduces a novel, holistic, and reproducible analytical framework integrating PCA and GIS to improve diagnostic accuracy and source identification. The findings are expected to inform sustainable groundwater governance at local and regional scales and support the development of evidence-based protection strategies amid rising anthropogenic pressures and climate induced water scarcity.

## Materials and methods

### Study area

**Fig. 1:**
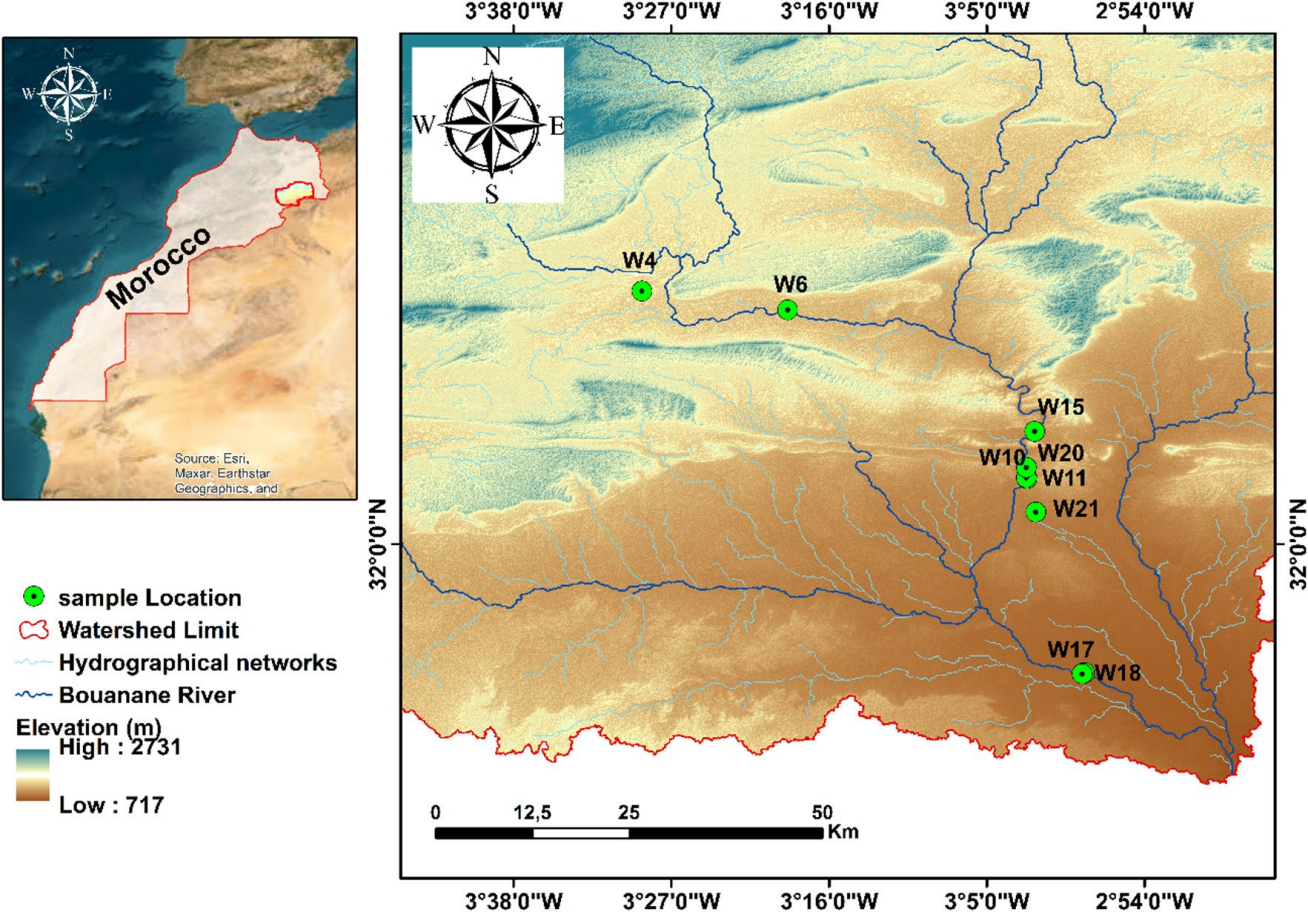
Geographic location of the study area and groundwater sampling wells. Satellite imagery was obtained from the Esri World Imagery basemap, and the map layout and spatial analysis were generated using ArcGIS Desktop version 10.8 (Esri Inc., Redlands, CA, USA; https://www.esri.com).

The watershed area (Fig. 1), covering approximately 1200 km^2^, is located in south-eastern Morocco, within the Bouanane watershed, which is itself part of the Guir watershed.

It is generally characterised by cold winters and hot summers, with low rainfall that is generally poorly distributed in terms of time and space^42^. The local economy is mainly based on agro-pastoral activities, in particular date palm cultivation and livestock farming in the surrounding oases. According to topographical data, Bouanane is located approximately at 32.0365° north latitude and − 3.0494° west longitude, at an average altitude of 950 m. Geomorphologically, the territory has a notable variation in altitude, ranging from 521 m to 2,731 m. The Bouanane basin is crossed by two main watercourses: the Oued Guir and the Oued Bouanane. The latter has an almost permanent flow, sustained by resurgences from multiple sources that compensate for water withdrawals for irrigation. The Oued Ait Aïssa, the main tributary of the Oued Bouanane, shares similar hydrological characteristics with the Upper Guir, also benefiting from significant inflows from perennial springs. The confluence of the Guir and Bouanane wadis is located in the Errachidia–Boudnib basin. In terms of climate, the region is characterised by a semi-arid climate, accentuated by its location on the edge of steep mountainous terrain. The hydrological regime of the watercourses is intermittent, with flows mainly linked to occasional floods. Average monthly temperatures vary between 11 °C and 40 °C, while average annual rainfall is around 119.8 mm. The region is also subject to frequent, sometimes violent winds, reaching speeds of over 100 km/h. Geomorphologically, the study area belongs to the middle zone of the Moroccan High Atlas. It is dominated by geological formations from the Jurassic and Cretaceous periods, strongly influenced by Alpine tectonics. The landscape is characterised by a succession of subparallel rocky ridges, oriented broadly along a west-east axis^43^. The upper basins of the Bouanane and Guir wadis comprise two distinct major aquifers, separated by an impermeable layer from the Toarcian-Aalenian period, as well as a system of Quaternary groundwater tables. The Lower Lias forms a continuous and highly productive fissured network, while the Aalenian (Dogger) series aquifers occur as fragmented synclinal basins. The Quaternary aquifers occupy the bottoms of transverse valleys and longitudinal depressions and are fed by karst networks and surface water infiltration^44^. In addition, the Cretaceous basin of Errachidia, located between the High and Anti-Atlas Mountains, contains two main aquifers: the Senonian aquifer, exploited via wells and boreholes and exhibiting strong artesianism, and the Turonian limestone aquifer^44^.

**Fig. 2:**
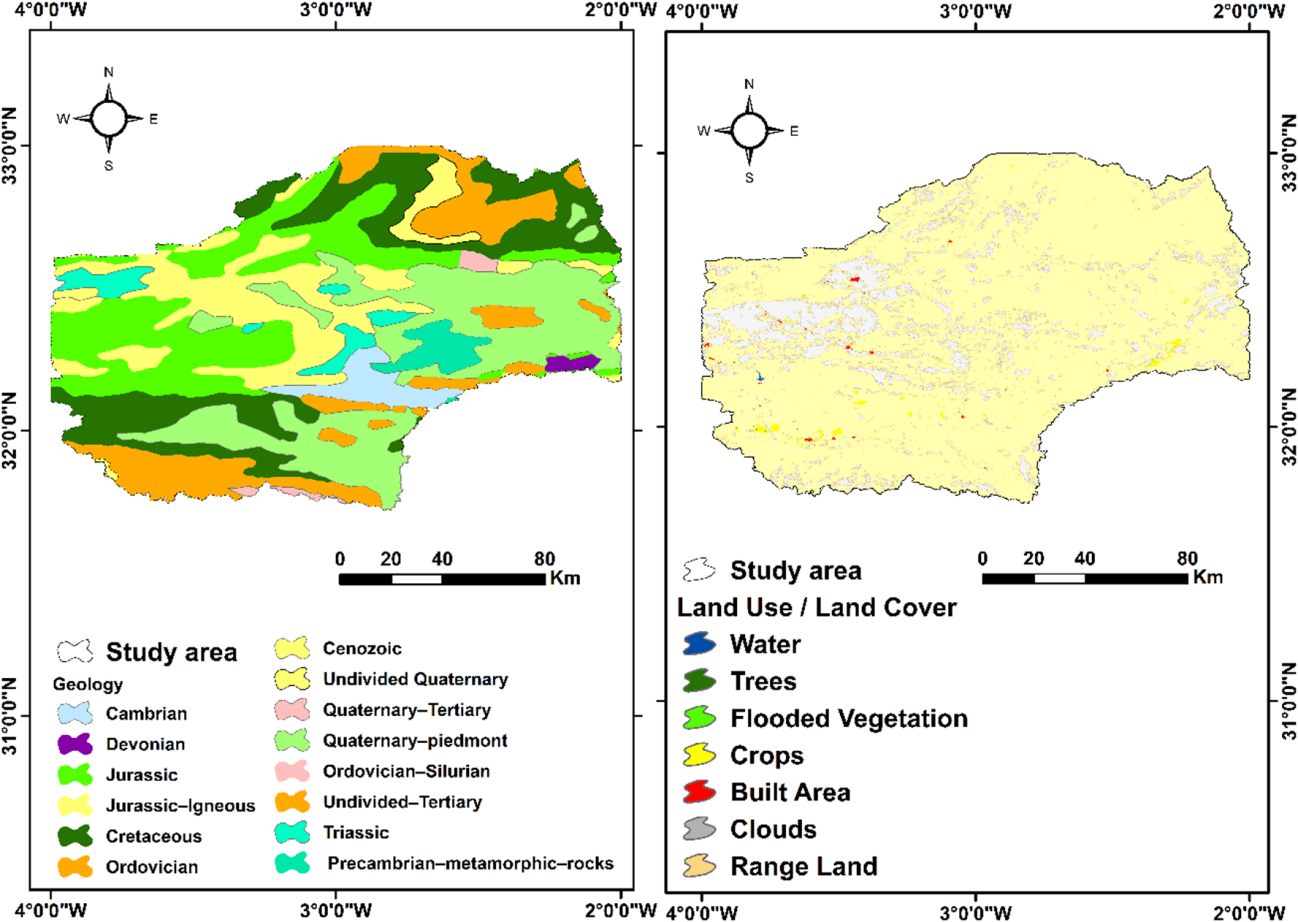
Geologic carte of the Bouanane watershed.

Geologically (Fig. 2), the study basin is characterised by stratigraphic diversity covering the periods from the Palaeozoic to the Quaternary^45^.

The Palaeozoic is marked by marine and continental sedimentary series composed mainly of schists, quartzites, sandstones and marine limestones^46^. The Ordovician period is represented by deposits of shale, sandstone and limestone reflecting a shallow marine environment, while the Devonian period shows evidence of deeper marine conditions, with limestone, marl, shale and sandstone intercalations.

The Mesozoic era, strongly influenced by Atlas tectonics, comprises Jurassic and Cretaceous formations structured into large synclines separated by major west-east-trending faults^43^. The Lower to Middle Triassic is characterised by red continental deposits (sandstone and mudstone). The Lower to Middle Jurassic is mainly calcareous-dolomitic, with a permeable and continuously extended Lower Lias. The Upper Jurassic is dominated by red-facies continental sandstone sediments^45^. The Cretaceous shows an alternation of sandstone, marl and limestone, reflecting marine and continental environments linked to the opening of the Atlas Basin^47^.

The Cenozoic era is characterised by continental sedimentary deposits in intramountain basins^48^. The Tertiary period consists mainly of limestone, more or less consolidated sandstone-limestone and soft purplish white sandstone. The Quaternary period includes recent alluvial deposits, alluvial fans and colluvial formations, associated with climatic cycles alternating between wet and dry periods.

Spatial land cover plays a vital impact in water quality. This characteristic delivers crucial details on the moisture content of soil, penetration processes, groundwater and surface water, although aiding to determine groundwater requirements^49,50^. For Bouanane watershed, 7 types of LULC were recognised, which includes water bodies, shrubs, vegetation, crops, built-up areas, barren land and pastures (Fig. 2).

### Sampling and laboratory analysis

Nine samples in Bouanane watershed have been obtained in April 2024 from representative monitoring stations distributed across the study area, including wells, springs, and underflow discharge zones (Fig. 2). To remove stagnant water and ensure that the samples reflected the true hydrochemical signature of the aquifer, each sampling point was purged by continuous pumping for about 10–15 min before collection. Samples were stored in pre-treated polypropylene bottles (1 L and 250 mL) that had been rinsed twice with distilled water and subsequently conserved for 24 h in a 10% hydrochloric acid (HCl) solution. This stringent pre-conditioning procedure minimized external contamination and ensured preservation of sample integrity for subsequent hydrochemical analyses. Immediately after collection, the water samples were stored in ice-cooled container and maintained at 4 °C to suppress chemical alteration and microbial activity during transport to the laboratory.

To enhance representativeness and capture conditions consistent with groundwater abstraction practices, sampling was conducted during active pumping of the boreholes. In situ physical properties, electrical conductivity, pH, total dissolved solids, and temperature, have been determined directly at each site using a multi parameter probe (HI 9829). These real time field measurements provided essential baseline information on the immediate hydrochemical state of the groundwater and formed a critical component of the validation process.

Comprehensive laboratory analyses were performed at AFRILAB Laboratory in Marrakesh. Principal cations (Ca^2+^, Mg^2+^, K^+^) and anions (SO₄²⁻, Cl⁻) have been measured employing Inductively Coupled Plasma–Optical Emission Spectrometry (ICP-OES). Bicarbonate (HCO₃⁻) values have been assessed by acid titration with sulphuric acid (H₂SO₄), following standard procedures outlined by^51^ and^52^. All ionic concentrations were expressed in mg/L. Analytical accuracy and dataset reliability were evaluated use the ion Balance ion (CBE), computed according to Eq. (1), and only results with a balance error within ± 5% were retained^53^. This QA/QC protocol ensured the robustness and reproducibility of the hydrochemical dataset.

1 $$\mathbf{C}\mathbf{B}\mathbf{E}=\frac{(\sum\mathbf{C}\mathbf{a}\mathbf{t}\mathbf{i}\mathbf{o}\mathbf{n}\mathbf{s}-\sum\mathbf{A}\mathbf{n}\mathbf{i}\mathbf{o}\mathbf{n}\mathbf{s})}{(\sum\mathbf{C}\mathbf{a}\mathbf{t}\mathbf{i}\mathbf{o}\mathbf{n}\mathbf{s}+\sum\mathbf{A}\mathbf{n}\mathbf{i}\mathbf{o}\mathbf{n}\mathbf{s})}\times100$$

A microbiological assessment was carried out in 2024 on the same nine groundwater samples previously selected, with the aim of detecting faecal contamination indicators. The investigation targeted four microbial groups: total coliforms, faecal coliforms, intestinal enterococci, and Escherichia coli.

All analytical steps adhered to the requirements of the Moroccan quality of water for drinking regulation^54^. For each sample, 100 mL of water were filtered through a 0.45-µm cellulose-ester membrane designed to retain the microorganisms of interest. Following filtration, the membranes were placed onto selective culture media and incubated at the appropriate temperatures specified for each indicator (Table 1).

After incubation, developed colonies were enumerated and results expressed as colony forming units per 100 mL. Each measurement was performed in triplicate to ensure analytical robustness, precision, and reproducibility.

It is vital to emphasize that GPS technology played a central role in precisely collecting the coordinates of each sampling site, hence providing precise location data. The gadget utilised was a Garmin GPSMAP^®^ 67i.

**Table 1:** Bacteriological analysis methods^21,54^.

Parameter	MSMAV: (CFU/100 mL)	WHO (2017) (CFU/100 mL)	Incubation temperature	Culture media
FC (ISO 9308-1)	0	0	44 °C	Tergitol 7 + TTC agar
TC (ISO 9308-1)	0	0	37 °C	Tergitol 7 + TTC agar
*Escherichia coli* (ISO 9308-1)	0	0	37 °C	MacConkey agar
IE (ISO 7899-2) – Intestinal *Enterococci*	0	0	37 °C	Slanetz and Bartley agar

### Pollution index of groundwater (PIG)

The evaluation of groundwater quality through the Groundwater Pollution Index (GPI) followed a standardised five stage procedure, similar with methodological frameworks extensively applied in recent research^24,26,55^.

In the first stage, each selected water quality criterion was assigned a relative weight (Rw), signifying its significance in judging drinking water acceptability. The weights, spanning from 1 to 5 (Table 2), show the magnitude of influence of each parameter higher values signal more significance in determining groundwater quality and its implications on human health. This weighting technique generates a hierarchical categorization that prioritizes parameters according to their potential impact on overall water quality.

In the second stage, the weighting coefficient (Wp) for each parameter was obtained by normalising the given Rw values through division by the cumulative sum of all relative weights (Eq. 2). This coefficient represents the proportional contribution of each parameter to the total index, demonstrating its relative importance inside the GPI computational framework.

2 $$\mathbf{W}\mathbf{p}=\frac{\mathrm{R}\mathrm{w}}{\sum\left(\mathrm{R}\mathrm{w}\right)}$$

The final stage consisted of determining the Specific Contamination Index (SOC) for each ion measured in the groundwater samples. This index was developed by comparing the observed concentration (Ci) to the respective drinking water guideline value (DWHO), as indicated in Eq. 3^1^. This calculation offers a direct estimate of the extent to which each ion approaches or exceeds its recommended threshold, so highlighting its individual contribution to possible groundwater quality deterioration.

3 $$\mathbf{S}\mathbf{O}\mathbf{C}=\frac{\mathrm{C}\mathrm{n}\mathrm{i}}{\mathrm{D}\mathrm{W}\mathrm{H}\mathrm{O}}$$

In the fourth phase, the overall groundwater quality (OQG) associated with each parameter was estimated by multiplying its weighting coefficient (Wp) by the accompanying Specific Contamination Index (SOC), as stated in Eq. 4. The GPI evaluation was then concluded by summing all OQG values for each sample, in line with Eq. 5. This aggregation gives an integrated signal that indicates the cumulative pollution load influencing the groundwater system.

**Table 2:** The parameters utilized in the computation of the groundwater pollution index (GPI).

Water quality variable	Relative weight (Rw)	Weight parameter (Rw)	Drinking water quality standards^21^
pH	5	0.1315	7.5
TDS (mg/L)	5	0.1315	500
TH (mg/L)	2	0.0526	300
Ca^2+^ (mg/L)	2	0.0526	75
Mg^2+^ (mg/L)	2	0.0526	30
Na⁺ (mg/L)	4	0.1052	200
K⁺ (mg/L)	1	0.0263	12
HCO₃⁻ (mg/L)	3	0.0789	300
Cl⁻ (mg/L)	4	0.1052	250
SO₄²⁻ (mg/L)	5	0.1315	200
NO₃⁻ (mg/L)	5	0.1315	50
	∑Rw = 38	∑Rw = 1	

4 $$\mathbf{O}\mathbf{Q}\mathbf{G}=\mathrm{W}\mathrm{p}\times\mathrm{S}\mathrm{O}\mathrm{C}$$

5 $$\mathbf{G}\mathbf{P}\mathbf{I}=\sum\mathrm{O}\mathrm{Q}\mathrm{G}$$

Groundwater quality classification was carried out using the GPI values, which sort groundwater into five pollution groups, ranging from low contamination to very high pollution. This classification system provides a uniform and trustworthy framework for understanding groundwater quality situations, enabling clearer geographic and comparative assessments of pollution intensity. It also facilitates the identification of high priority regions requiring quick intervention, so increasing evidence based decision making for groundwater protection and sustainable management.

### Nitrate pollution assessment

Nitrate contamination is recognized as a major driver of groundwater pollution worldwide. To evaluate the extent of nitrate related degradation, the (NPI) is employed. The NPI is calculated using the formula provided in Eq. 6, offering a standardized measure to quantify the influence of nitrate concentrations on water quality.

6 $$\boldsymbol{N}\boldsymbol{P}\boldsymbol{I}=\frac{{C}_{m}-{C}_{s}}{{\mathrm{C}}_{s}}$$

The (NPI) is determined by comparing the measured nitrate concentration (Cm) in groundwater samples with a threshold value (Cs) established according to anthropogenic influences. A commonly recommended threshold of 10 mg/L is adopted for this purpose^56^. The resulting NPI values are then categorised into five distinct levels, each representing a specific pollution class, as summarized in Table 3^55^. This methodology provides a standardized assessment of nitrate impact on groundwater quality and enables the identification of areas requiring targeted management interventions.

**Table 3:** The category of the nitrate pollution index (NPI).

NPI value	NPI interpretation
< 0	Unpolluted
0–1	Low pollution
1–2	Moderate pollution
2–3	High pollution
> 3	Very significant pollution

### Nitrate human health risk assessment

Human populations receive exposure to contaminants through two main pathways: ingestion and dermal contact^57^. This study examines both approaches, with special attention on water used for drinking and domestic use. Risks were investigated by establishing the chronic daily intake (CDI), expressed as mg/kg/day, for adults and children. The method follows issued protocols^58^, which also quantified oral and dermal contact for these groups.

Determine the chronic daily quantity by oral ingesting from drinking water (Eqs. 7 and 8):

7 $${\boldsymbol{H}\boldsymbol{Q}}_{\boldsymbol{o}\boldsymbol{r}\boldsymbol{a}\boldsymbol{l}}=\frac{\mathrm{C}\mathrm{D}\mathrm{I}}{\mathrm{R}\mathrm{f}\mathrm{D}}$$

8 $$\boldsymbol{C}\boldsymbol{D}\boldsymbol{I}=\frac{\mathrm{C}\times\mathrm{I}\mathrm{R}\times\mathrm{E}\mathrm{D}\times\mathrm{E}\mathrm{F}}{\mathrm{B}\mathrm{W}\times\mathrm{A}\mathrm{T}}$$

Determine the hazard quotient for the consumption of non carcinogenic components by dermal contact at contaminated water (Eqs. 9 and 10):

9 $${\boldsymbol{H}\boldsymbol{Q}}_{\boldsymbol{d}\boldsymbol{e}\boldsymbol{r}\boldsymbol{m}\boldsymbol{a}\boldsymbol{l}}=\frac{\mathrm{C}\mathrm{D}\mathrm{D}}{\mathrm{R}\mathrm{f}\mathrm{D}}$$

10 $$\boldsymbol{C}\boldsymbol{D}\boldsymbol{D}=\frac{\mathrm{C}\times\mathrm{K}\times\mathrm{S}\mathrm{A}\times\mathrm{E}\mathrm{V}\times\mathrm{E}\mathrm{T}\times\mathrm{C}\mathrm{F}\times\mathrm{E}\mathrm{D}\times\mathrm{E}\mathrm{F}}{\mathrm{B}\mathrm{W}\times\mathrm{A}\mathrm{T}}$$

Determine the hazard index for the non carcinogenic risk of contaminants into the human by combined oral consumption and dermal contact (Eq. 11):

11 $$HI={HQ}_{dermal}+{HQ}_{oral}$$

In the HHRA model, hazard quotients (HQ) determine non carcinogenic risks through ingestion and dermal contact. The total hazard index (HI) is the sum of both. Daily levels are compared with reference values (RfD = 1.6 mg/kg/day for nitrates). Important standards are concentration, body weight, exposure duration, and skin permeability (Table 4). An HI above 1 suggests high risk; below 1 indicates low risk^59^.

**Table 4:** Characteristics for health risk assessment method.

Item	IR	K	EV	ET	CF	ED	EF	BW	AT	SA
Adults	1.5	0.001	1	0.4	0.001	365	30	70	10,950	17,920
Children	0.7	0.001	1	0.4	0.001	365	6	30	2,190	9,424

*IR* Ingestion Rate (L/day), *K* Skin Permeability Coefficient (cm/h), *EV* Exposure Event (days), *ET* Exposure time (h/event), *CF* Conversion Factor, *ED* Exposure Duration (Days), *EF* Exposure Frequency (days/year), *BW* Body Weight (kg), *AT* Averaging Time (days), *SA* Skin Surface Area (cm^2^).

### Geospatial assessment

ArcGIS was employed as a core geospatial platform for modelling, visualising, and predicting the spatial dispersion of pollutants in groundwater systems. Its advanced geoprocessing and spatial analysis capabilities enable the integration, interpretation, and visual representation of hydrochemical datasets, thereby strengthening evidence-based decision making in environmental management and land use planning. In this study, ArcGIS facilitated the generation of spatial distribution maps for key physicochemical parameters and groundwater pollution indices, providing a clear depiction of spatial heterogeneity and contamination patterns across the study area^60^. This geospatial approach offers a comprehensive understanding of contaminant dynamics, supports the assessment of current groundwater quality status, and enhances the ability to forecast potential pollution trajectories under varying anthropogenic and environmental pressures^61,62^. Spatial interpolation of groundwater quality variables was performed using the Inverse Distance Weighting (IDW) technique, selected for its effectiveness in representing spatial variability based on sampling density and proximity relationships.

The spatial outputs generated through ArcGIS provided a robust visual framework for identifying pollution hotspots, assessing variations in groundwater chemistry, and evaluating the spatial extent of quality degradation within the study area. Such geospatial insights are crucial for targeted groundwater protection, resource management, and the formulation of sustainable mitigation strategies.

**Fig. 3:**
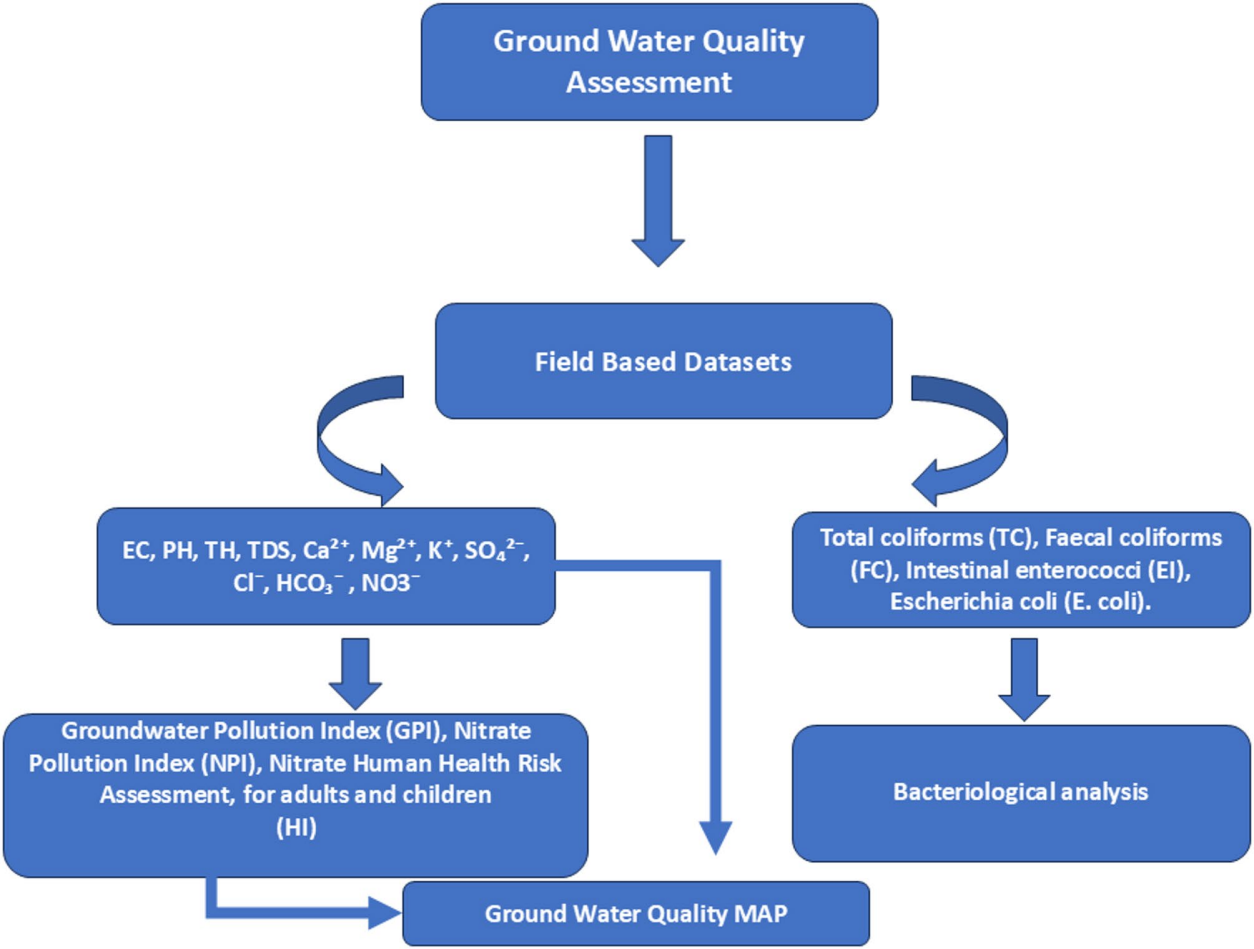
Adopted flowchart for groundwater quality assessment.

To integrate these different approaches into a comprehensive overview of the adopted methodology, Fig. 3 presents the methodological schematic illustrating the main steps of the study.

## Results

### General hydrochemical characteristics

**Fig. 4:**
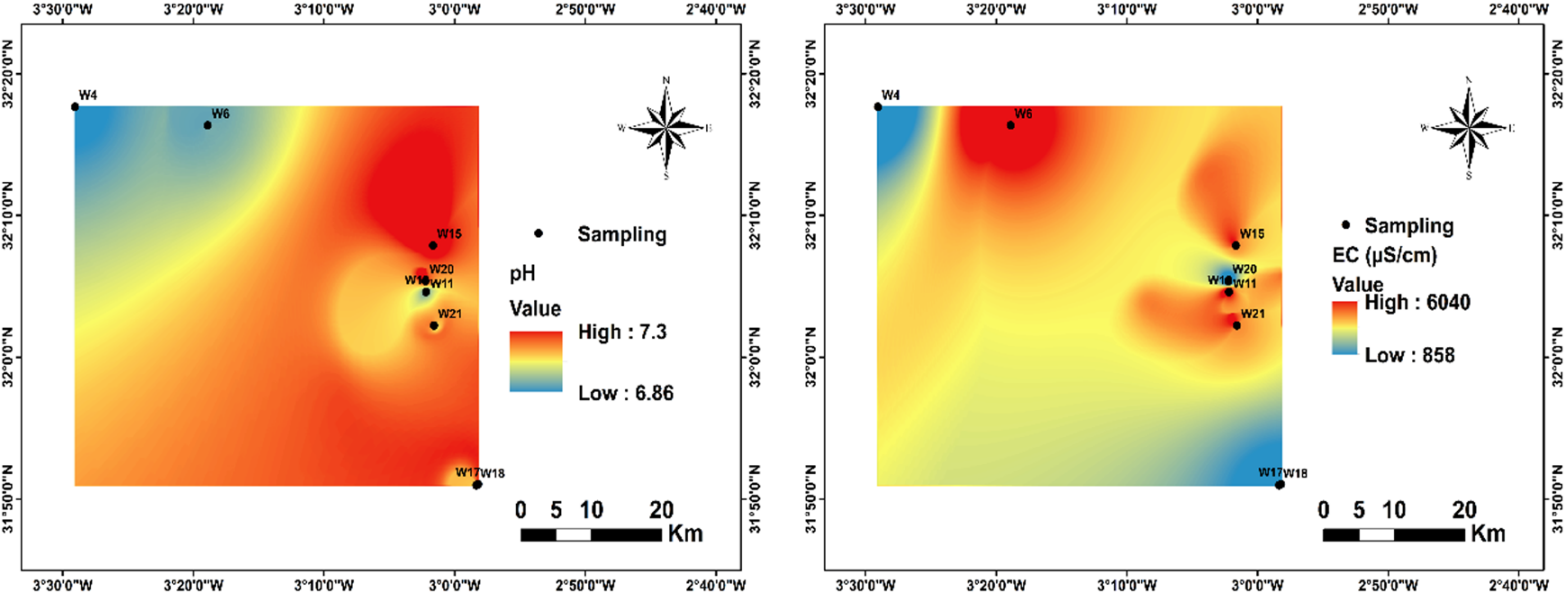
Spatial distribution of pH and CE in the region.

As part in this investigation, spatial groundwater quality mappings were developed using ArcGIS 10.8 to offer an extensive graphical illustration for the hydrochemical state of groundwater within the study area. These themed maps (Fig. 4) depict the regional variability of major water quality indices, enabling the detection of contaminated hotspots, hydrochemical gradients, and emerging spatial patterns. The evaluation of groundwater suitability for consumption was performed in conformity with the reference criteria established by the World Health Organization^21^, ensuring compliance with internationally recognised quality thresholds and facilitating a scientifically robust interpretation of the results. Ased on average values, the dominating anion is HCO₃⁻, followed by Cl⁻, SO₄²⁻, and NO₃⁻ (HCO₃⁻ > Cl⁻ > SO₄²⁻ > NO₃⁻). Regarding the cations, Ca²⁺ is the major ion, followed by Mg²⁺, and K⁺ (Ca²⁺ > Mg²⁺ > K⁺).

In this investigation, pH values varied between 6.86 and 7.30, having an average of 7.05, indicating predominantly neutral conditions (Fig. 4). All recorded values fall within the permissible limits for drinking water (6.5–8.5) established by the World Health Organization^1^, confirming the appropriateness of the groundwater for human consumption with respect to acidity–alkalinity balance.

In the present study, EC values ranged from 858 to 6040 µS/cm, with a mean of 3587.6 µS/cm, markedly exceeding the World Health Organization^1^ recommended threshold for drinking water (500–1500 µS/cm) (Fig. 4). Notably, approximately 66.7% of the sampled stations surpassed this limit, highlighting a widespread salinisation issue.

**Fig. 5:**
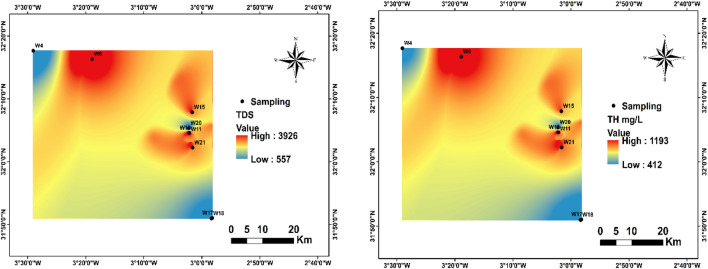
Spatial distribution of TDS and TH in the region.

TDS values varied between 557 and 3926 mg/L, having an average of 2332 mg/L, largely exceeding the^1^ drinking water recommendation of 500–1000 mg/L (Fig. 5). Around 66% of the sampled stations exceeded this limit, indicating high and widespread mineralization across the study area.

In this study, TH values ranged from 412 to 1193 mg/L, with an average of 824 mg/L, substantially exceeding the^1^ guideline for drinking water (100–500 mg/L) (Fig. 5). Nearly 88.9% of the sampling stations surpassed this threshold, confirming the presence of very hard water across the study area.

**Fig. 6:**
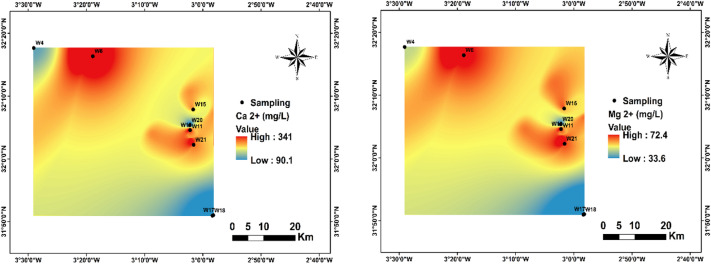
Spatial distribution of Ca^2+^ and Mg^2+^ in the region.

In this study, Ca²⁺ concentrations ranged from 90.1 to 340.84 mg/L, with an average of 215.2 mg/L. Approximately 55% of the sampling stations exceeded the^1^ guideline of 75–200 mg/L (Fig. 6). The highest concentrations were recorded at stations W6, W10, W11, and W15.

Mg²⁺ concentrations ranged from 33.61 to 72.4 mg/L, with an average of 57.6 mg/L, generally remaining below the^1^ guideline of 50–150 mg/L (Fig. 6).

**Fig. 7:**
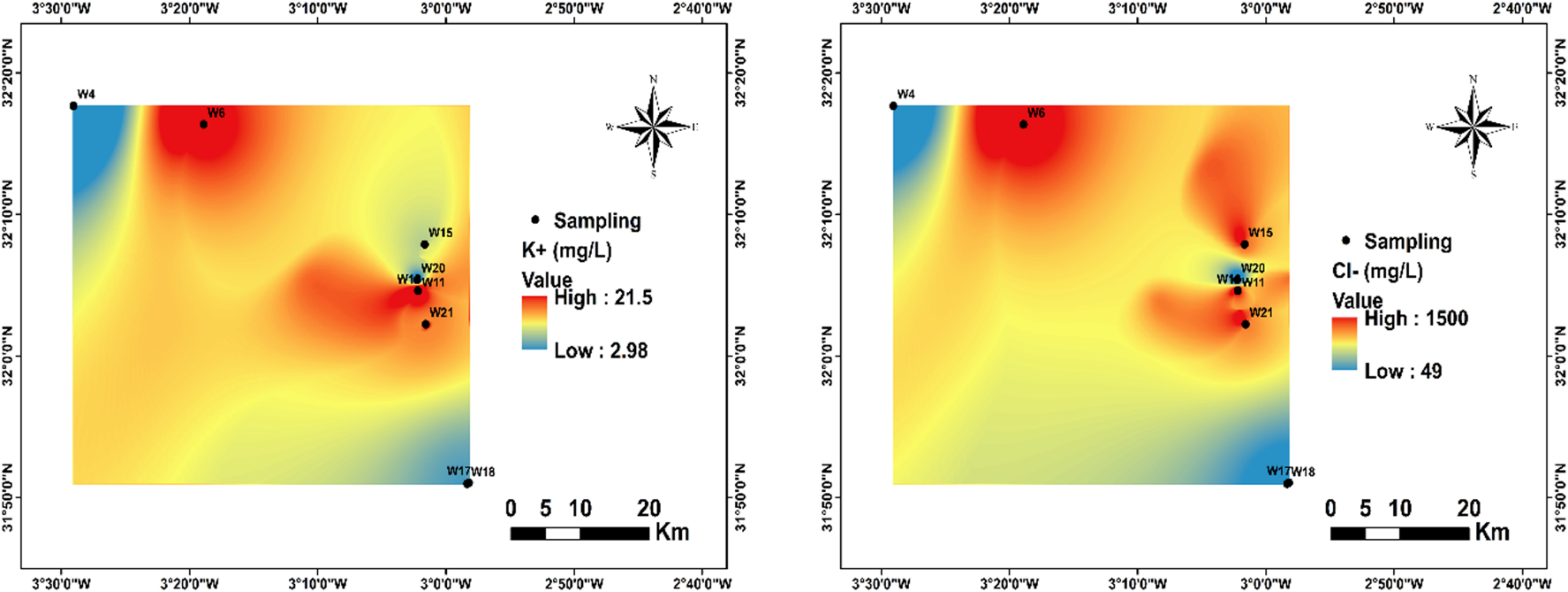
Spatial distribution of K^+^ and Cl^-^ in the region.

In this study, K⁺ concentrations varied between 2.98 and 21.5 mg/L, with an average of 11.8 mg/L. Approximately 44.4% of the sampling stations exceeded the^1^ guideline value of 12 mg/L (Fig. 7). The highest concentrations were recorded at stations W10, W11, W6, and W21.

Cl⁻ concentrations ranged from 49.52 to 1499.03 mg/L, with an average of 826.4 mg/L. Approximately 66% of the sampling stations exceeded the^1^ guideline of 200–600 mg/L (Fig. 7). The highest concentrations were recorded at stations W6, W10, W11, W15, and W21, mainly located in near downstream areas.

**Fig. 8:**
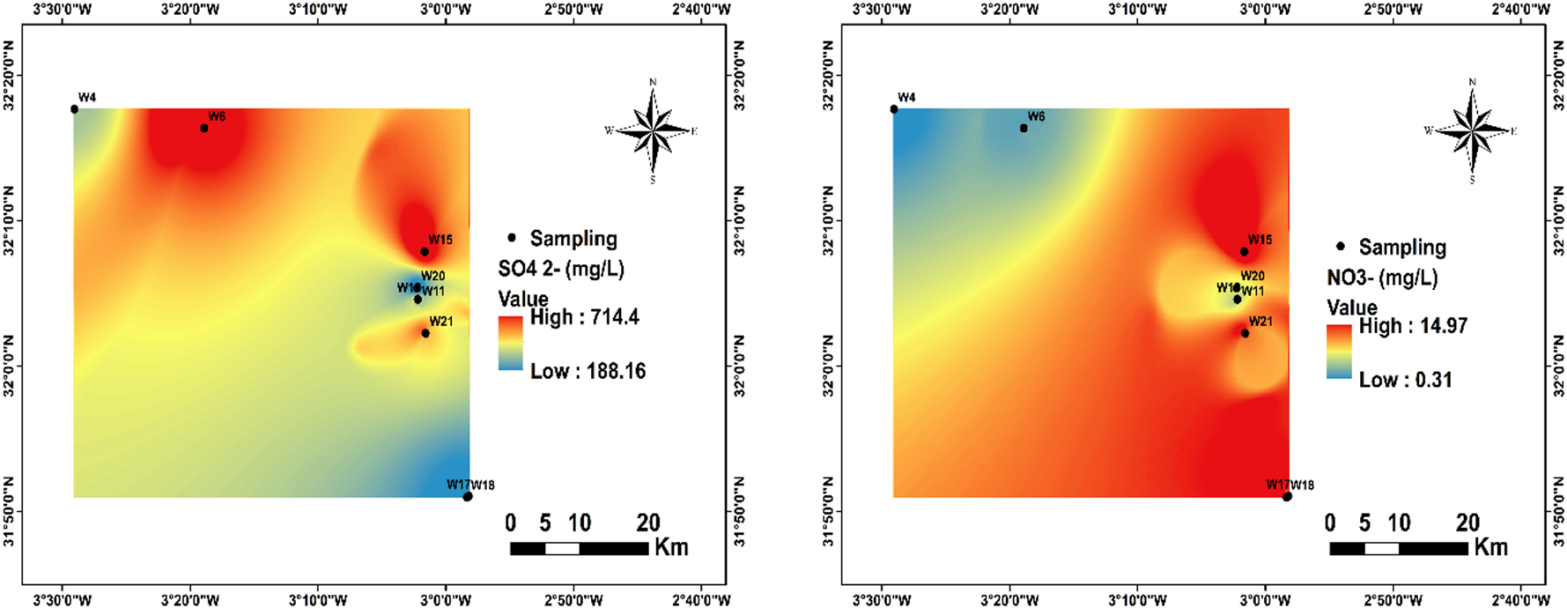
Spatial distribution of SO_4_^2-^ and NO_3_^-^in the region.

Sulfate ions (SO₄²⁻) in the groundwater of the Bouanane Basin ranged from 188.16 to 738 mg/L, with an average of 459.2 mg/L. Based on the^1^ guideline of 200–400 mg/L, approximately 55% of the sampling stations (W6, W10, W11, W15, W21) exceeded the upper limit, while 45% (W4, W17, W18, W20) remained within acceptable levels (Fig. 8). The highest concentrations were generally recorded at downstream stations. Nitrate (NO₃⁻) is a naturally occurring ion that plays a key role in the nitrogen cycle. However, chronic exposure to elevated nitrate concentrations in drinking water can lead to serious health effects, including methemoglobinemia (blue baby syndrome), digestive disorders, and complications for vulnerable groups, particularly infants and pregnant women^63,64^. In the Bouanane Basin, NO₃⁻ concentrations ranged from 0.31 to 14.97 mg/L, with an average of 6.83 mg/L, remaining well below the^1^ guideline value of 50 mg/L (Fig. 8). None of the sampling stations exceeded this limit.

**Fig. 9:**
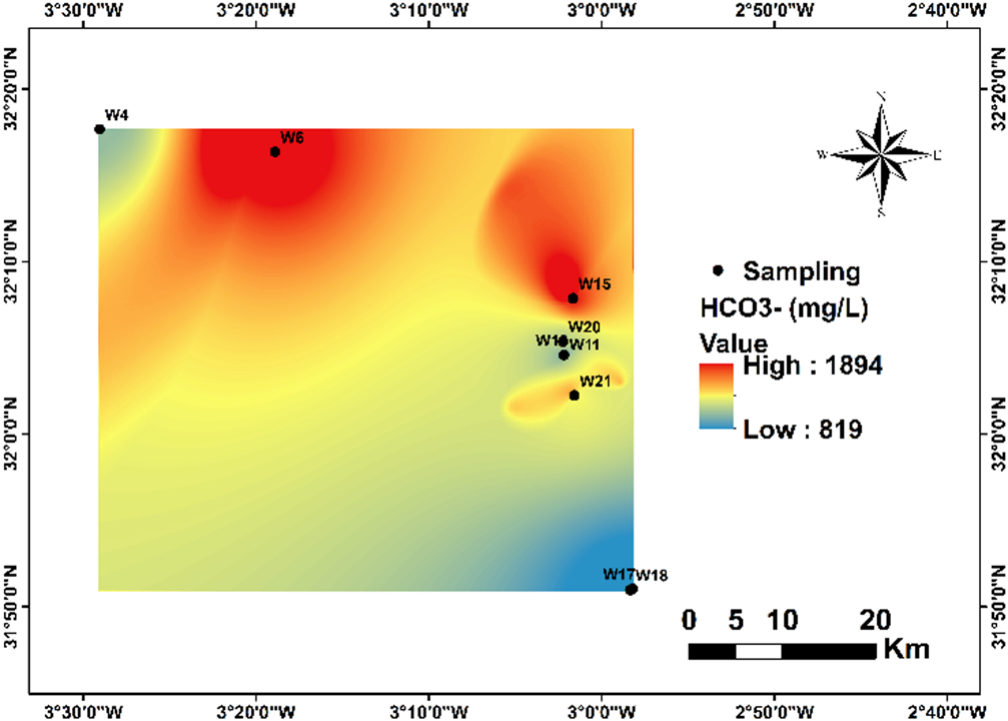
Spatial distribution of HCO_3_^-^ in the region.

Bicarbonate ions (HCO₃⁻) in the groundwater of the Bouanane Basin ranged from 819.23 to 1893.39 mg/L, with a mean of 1343.57 mg/L, substantially exceeding the^1^ guideline of 300–500 mg/L. All sampled stations (100%) recorded concentrations above this limit (Fig. 9).

### Water quality assessment using PIG

The groundwater quality assessment conducted in the Bouanane Basin using the (GPI) provides a comprehensive and integrative framework for synthesizing multiple physicochemical parameters into a single pollution indicator. The GPI values obtained reveal substantial spatial variability, reflecting the heterogeneous hydrogeochemical conditions across the aquifer system. The range of values (1.12–3.03) highlights pronounced differences in water quality levels and underscores the usefulness of GPI for identifying zones where geochemical processes exert the strongest influence.

**Fig. 10:**
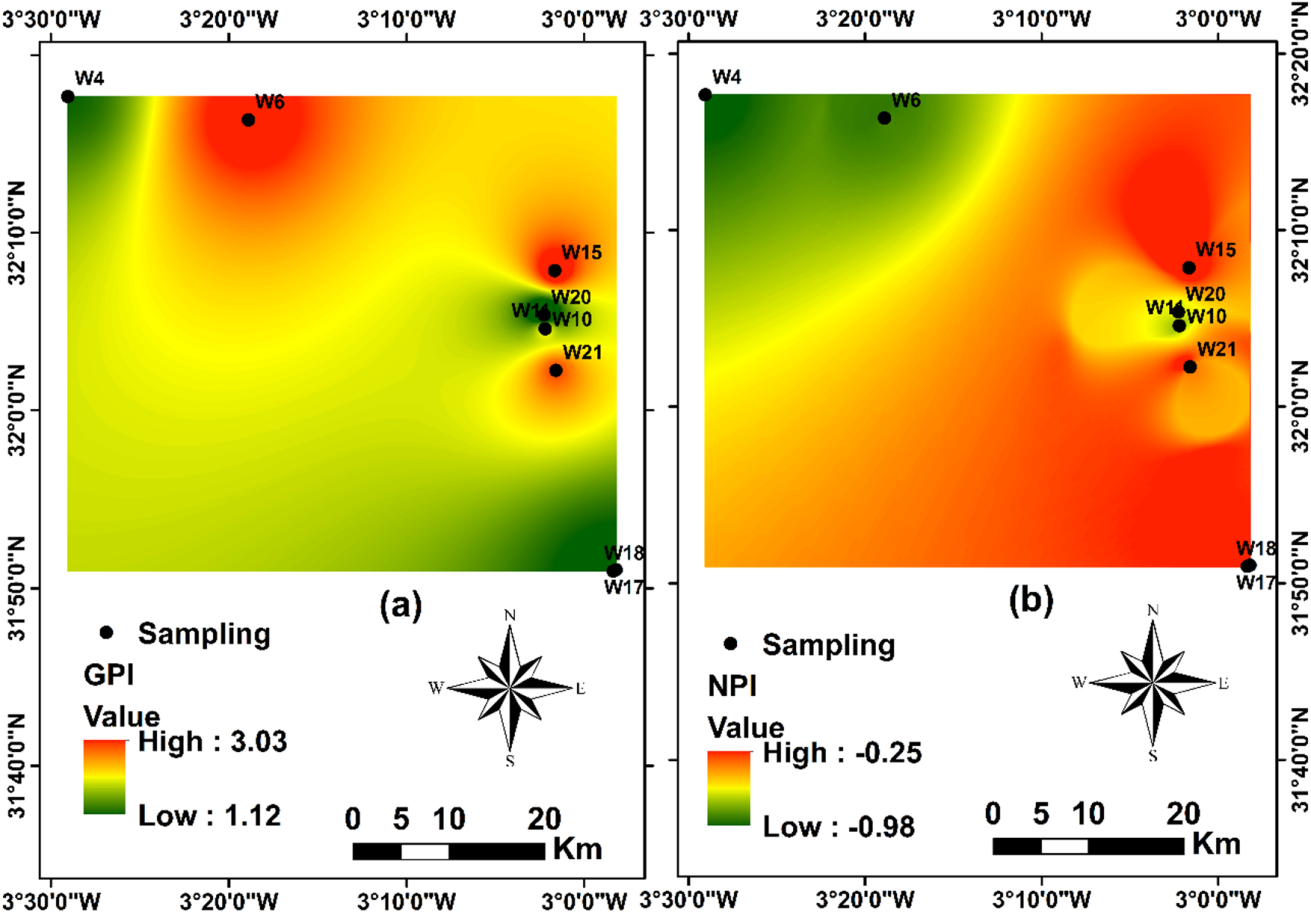
(**a**) Spatial Distribution of GPI and (**b**) Spatial Distribution of NPI.

The classification results indicate that none of the sampled stations fall within the “insignificant” category, suggesting that the entire aquifer system is affected albeit to varying degrees by detectable geochemical or anthropogenic influences. Low to moderate pollution levels, representing 44% of the stations, correspond to areas where mineralization remains relatively limited. Conversely, nearly half of the sampling points (44%) display very high pollution levels, pointing to intensified geochemical dissolution processes or hydrodynamic conditions that favor the accumulation of dissolved ions (Table 5; Fig. 10a).

**Table 5:** The category of the groundwater pollution index (GPI) for this study area.

Pollution category	GPI range	Stations	Number of stations	Percentage (%)
Insignificant	< 1	–	0	0%
Low	1–1.5	W18, W20	2	22%
Moderate	1.5–2	W4, W17	2	22%
High	2–2.5	W11	1	11%
Very High	> 2.5	W6, W10, W15, W21	4	44%

### Nitrate pollution analysis

The application of the (NPI) in the Bouanane Basin provides a reliable framework for evaluating the extent to which human derived nitrate sources may influence groundwater quality. The values obtained, ranging from − 0.98 to − 0.25, fall well below the established pollution threshold and thus confirm the absence of significant nitrate enrichment within the aquifer system. This narrow and consistently negative interval reflects minimal anthropogenic impact across the study area (Fig. 10b).

According to the standard NPI classification, all sampling stations are categorized as “unpolluted,” indicating that typical anthropogenic nitrate sources such as nitrogen based fertilizers, domestic effluents, or livestock waste play only a minor role in the current hydrochemical regime. Several environmental and socio-economic factors likely contribute to these low nitrate levels: (i) low intensity agricultural practices that limit fertilizer application; (ii) reduced rainfall during the study period, which curtails soil leaching and nitrate transport to deeper horizons; and (iii) the natural self purification capacity of soils and aquifer materials through adsorption, ion exchange, or microbially mediated denitrification.

### Human health risk assessment (HHRA)

Human Health Risk Assessment (HHRA) provides a comprehensive framework for quantifying the potential health impacts of contaminants through the two dominant exposure pathways: ingestion and dermal absorption, following the methodological guidelines outlined by^57^. In the Bouanane Basin, non-carcinogenic Hazard Index (HI) values ranged from 0.01 to 0.48 for adults and 0.01 to 0.40 for children, with mean values of approximately 0.21 and 0.18. These consistently low values, all well below the safety threshold (HI < 1), demonstrate that nitrate exposure from groundwater does not pose a significant health risk to local populations.

The highest HI values were recorded at stations W15, W17, W18, and W21, all located in the downstream portion of the basin. This area is characterized by more intensive agricultural activity, which may contribute to localized nitrate enrichment through fertilizer application and leaching processes. Agricultural return flow and soil nitrogen mobilization likely represent the dominant mechanisms controlling nitrate distribution. Nevertheless, even in these relatively more impacted areas, HI values remain well below the established risk threshold, indicating minimal human health concern at present.

The ingestion pathway overwhelmingly dominates total exposure, while the dermal route contributes only negligibly. This pattern aligns with global hydro-epidemiological findings indicating that drinking water ingestion represents the primary mechanism for nitrate transfer to the human body^59^. Children consistently exhibit higher HI values than adults due to their lower body weight and relatively higher water intake per unit of body mass factors that increase their susceptibility, as documented by^58^. Despite this heightened vulnerability, all calculated hazard indices remain comfortably within safe limits.

**Fig. 11:**
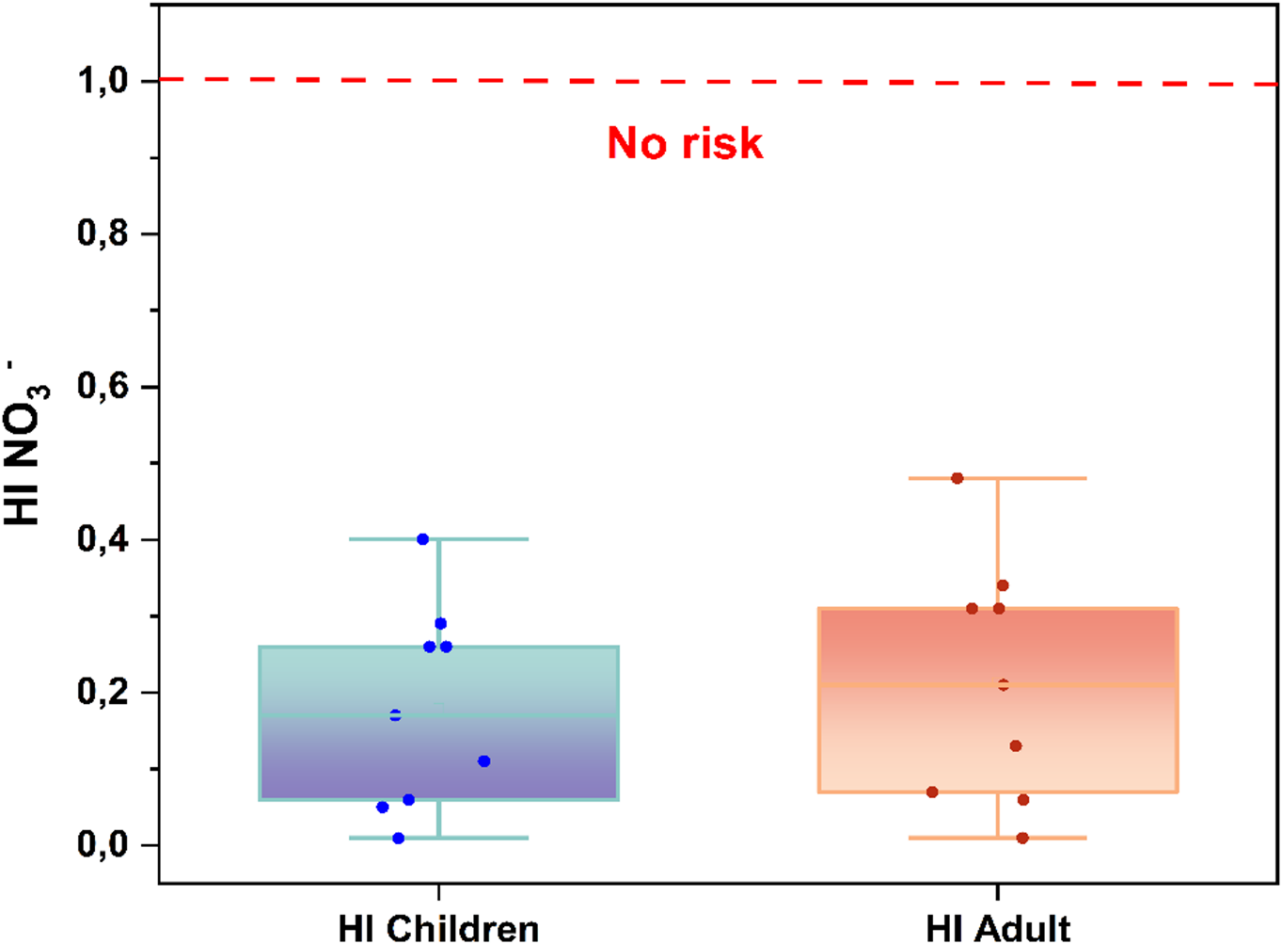
Results of HI for children and adults.

These results are consistent with observations from other Moroccan basins characterized by limited nitrate pollution^65^, further reinforcing the conclusion that nitrate related health risks in the Bouanane Basin are minimal. Overall, the combined evidence indicates that natural hydrogeochemical processes, together with moderate anthropogenic influence, maintain groundwater nitrate concentrations at levels that do not pose a significant threat to human health (Fig. 11).

## Discussion

### Hydrochemical variation

A clear spatial gradient in pH was observed across the basin, with comparatively higher values recorded at downstream stations relative to upstream sites. This increase is consistent with progressive carbonate mineral dissolution along the groundwater flow path, which buffers acidity and stabilizes pH under alkaline conditions. In contrast, the slightly lower pH values measured at upstream stations (W4 and W6) likely reflect higher CO₂ availability derived from soil respiration, surface infiltration processes, and localized lithological heterogeneity that modulates aquifer geochemistry.

Maintaining pH within recommended limits is essential for drinking water quality and infrastructure protection. Acidic conditions can promote metal mobilization and pipe corrosion, whereas highly alkaline waters may alter nutrient solubility and biological availability. The predominantly neutral conditions observed in the study area indicate a well-buffered carbonate system with limited anthropogenic disturbance, consistent with previous findings reported by^66^.

Electrical conductivity exhibited a marked downstream increase, with the highest values recorded at W10, W11, and W15. This pattern reflects the cumulative acquisition of dissolved ions along the groundwater flow path. The anomalously high EC value at upstream station W6 suggests localized interaction with highly soluble lithological units, particularly evaporitic deposits. Elevated EC therefore indicates advanced mineralization processes that directly influence groundwater potability.

A comparable spatial trend was observed for TDS. Lower concentrations at W4 and certain downstream stations (W20 and W18) contrast with pronounced enrichment at W10, W11, W15, W21, and W6. This heterogeneity primarily reflects spatial variability in lithology and localized anthropogenic influences, particularly agricultural return flows and surface-derived inputs. Elevated TDS levels at specific stations may be associated with enhanced mineral dissolution from host rock formations combined with irrigation-induced salinization processes. Similar patterns have been documented in intensively cultivated regions, where TDS enrichment has been linked to agricultural practices and soil salinity buildup^67^. Elevated TDS levels may reduce palatability and exert laxative effects, particularly among individuals with kidney disorders^21^.

Total hardness values were highest in areas where groundwater interacts with calcium- and magnesium-rich formations, underscoring the dominant influence of carbonate and silicate mineral dissolution. Anthropogenic inputs may exert a secondary influence. High hardness reduces soap efficiency and increases boiling point of water^17^. while concentrations exceeding 300 mg/L have been associated with potential cardiovascular and renal complications^17^. Based on^22^ classification, the groundwater in the study area exhibits hay level of hardness.

Calcium and magnesium represent the principal contributors to hardness and originate primarily from the dissolution of carbonate minerals such as calcite, dolomite, and and gypsum^1^. The observed dominance of calcium over magnesium aligns with the geochemical behavior of carbonate aquifers, where calcite and gypsum dissolve more readily than dolomite, resulting in preferential Ca²⁺ enrichment^67^.

Overall, geological controls constitute the primary source of Ca²⁺ and Mg²⁺ in the aquifer, although minor anthropogenic contributions from fertilizers and surface water infiltration cannot be excluded^68,69^. Elevated calcium concentrations may affect taste and induce digestive discomfort in sensitive individuals, whereas magnesium mainly contributes to overall hardness and domestic usability. Potassium concentrations reflect the weathering of feldspars, micas, and clay minerals within the aquifer matrix. Given its relatively high solubility, K⁺ is progressively released during mineral alteration processes. Elevated values therefore largely mirror potassium-rich lithology, although agricultural inputs and surface water infiltration may provide additional contributions. While potassium is generally safe at moderate concentrations, excessive intake may disrupt electrolyte balance and contribute to cardiovascular complications in individuals with kidney disorders^15^. Therefore, stations showing elevated K⁺ concentrations should be regularly monitored to ensure drinking water safety^70,71^.

Chloride enrichment follows a spatial pattern indicative of progressive mineralization along the groundwater flow path. Although chloride may originate from natural dissolution of chloride-bearing minerals or seawater intrusion, anthropogenic inputs such as irrigation return flows may enhance local concentration^72,73^. Elevated chloride adversely affects organoleptic properties, increases corrosivity, and has been associated with hypertension and cardiovascular complications under prolonged exposure^74^. he distribution observed in this study suggests that geological controls dominate, while human influence remains secondary.

Sulfate enrichment similarly reflects prolonged groundwater–rock interaction and dissolution of evaporitic deposits within carbonate–dolomitic formations. Although geogenic processes appear predominant, localized irrigation return flows and surface water infiltration may contribute to elevated SO₄²⁻ levels. High sulfate concentrations impart a bitter taste and may produce laxative effects, diarrhea, and dehydration^16,72^. These results highlight the need for regular monitoring to ensure the suitability of groundwater for drinking purposes. The spatial distribution of nitrate indicates limited anthropogenic impact despite the presence of agriculture and septic systems. This suggests that current land-use practices have not yet resulted in significant nitrate accumulation within the aquifer. Nevertheless, proactive monitoring remains essential to prevent future contamination.

Bicarbonate enrichment is primarily controlled by prolonged interaction between groundwater and carbonate-rich formations, including limestone, dolomite, and evaporitic horizons. Carbonic acid (H₂CO₃), formed from atmospheric CO₂, root respiration, and microbial processes, enhances mineral dissolution and releases bicarbonate into the aquifer system^75^. Elevated HCO₃⁻ concentrations increase alkalinity, contribute to pH stabilization, and may promote scaling in distribution systems. These findings emphasize the long-term geochemical evolution of the aquifer and highlight the importance of sustained monitoring to safeguard drinking water quality.

### Pollution index assessment

The spatial distribution of the Groundwater Pollution Index (GPI) exhibits a pronounced correspondence with zones of elevated electrical conductivity (EC), revealing a coherent hydrogeochemical structure controlled primarily by lithology (Fig. 10). Areas characterized by high GPI values systematically coincide with sectors enriched in dissolved ions, reflecting intensified mineralization processes. The dominance of Ca²⁺, Mg²⁺, HCO₃⁻, Cl⁻, and SO₄²⁻ unequivocally indicates the dissolution of calcite, dolomite, gypsum, and halite derived from the carbonate–evaporitic formations of the basin. This geochemical coherence confirms that groundwater quality degradation in the Bouanane Basin is fundamentally governed by water–rock interactions rather than external contamination sources.

**Fig. 12:**
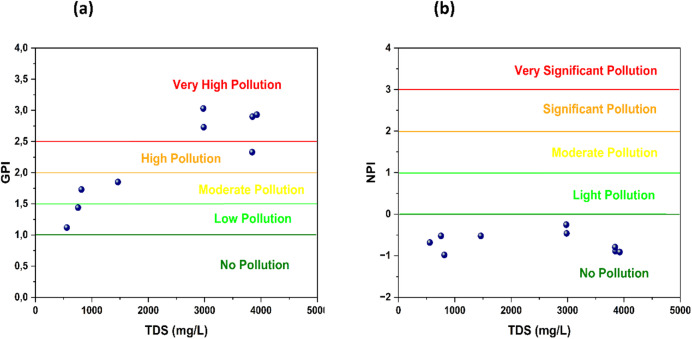
(**a**) Scatter plots of GPI versus TDS and (**b**) Scatter plots of NPI versus TDS.

The strong positive relationship between GPI and total dissolved solids (TDS) further validates this interpretation (Fig. 12a). All stations categorized as polluted or highly polluted exceed the 1000 mg/L TDS threshold; however, this exceedance reflects natural mineral enrichment instead of anthropogenic inputs. The ability of GPI to accurately capture both the magnitude and spatial organization of mineralization processes demonstrates its reliability as an integrative hydrochemical assessment tool. In quantitative terms, the majority of groundwater samples fall within the low to moderate pollution classes, while only a limited proportion reaches the high pollution category, underscoring that the observed deterioration is predominantly lithogenic in origin.

The (NPI) results reinforce this conclusion. Nitrate concentrations comply with international drinking-water standards across all sampling points, and NPI values predominantly indicate insignificant to low nitrogen pollution (Fig. 12b). Importantly, several stations display elevated TDS while maintaining low nitrate levels, demonstrating a clear decoupling between mineralization and anthropogenic nitrogen inputs. This divergence provides compelling evidence that groundwater mineralization is unrelated to agricultural or urban nitrate contamination and instead reflects the dissolution of carbonate and evaporitic minerals within the aquifer matrix.

When compared with recent regional studies, the hydrochemical behavior of the Bouanane Basin reveals distinctive characteristics. In^76^ reported that 28% of samples exhibited insignificant pollution, 48% low pollution, and 24% high pollution according to PIG values, while NPI results indicated 24% insignificant, 44% mild, and 32% moderate nitrogen pollution, highlighting a mixed influence of natural processes and anthropogenic pressures. In contrast, the present study shows a markedly lower proportion of nitrogen-related pollution (IPN) and a clearer dominance of lithogenic control, indicating limited agricultural or urban impact within the basin.

Similarly^77^, found that 44.44% of waters were suitable for drinking, 50% were of medium quality, and 5.56% were unsuitable, with hydrochemical facies dominated by Ca–Mg–Cl and Ca–Cl types and significant anthropogenic influence superimposed on water–rock interaction processes. By comparison, the Bouanane Basin exhibits mineralization patterns strongly aligned with carbonate–evaporite dissolution, while anthropogenic signatures remain weak, as evidenced by consistently low IPN values.

Overall, the integrated GPI–IPN assessment demonstrates that groundwater chemistry in the Bouanane Basin is overwhelmingly controlled by geological processes. The clear spatial coupling between GPI, EC, and TDS, combined with the systematic compliance of nitrate concentrations with drinking-water standards, confirms that mineralization is lithogenically driven and largely independent of human-induced nitrogen contamination. This distinction not only strengthens the robustness of the applied indices but also positions the present study as a rigorous hydrogeochemical assessment that successfully differentiates natural mineralization from anthropogenic pollution.

### Correlation analysis

**Fig. 13:**
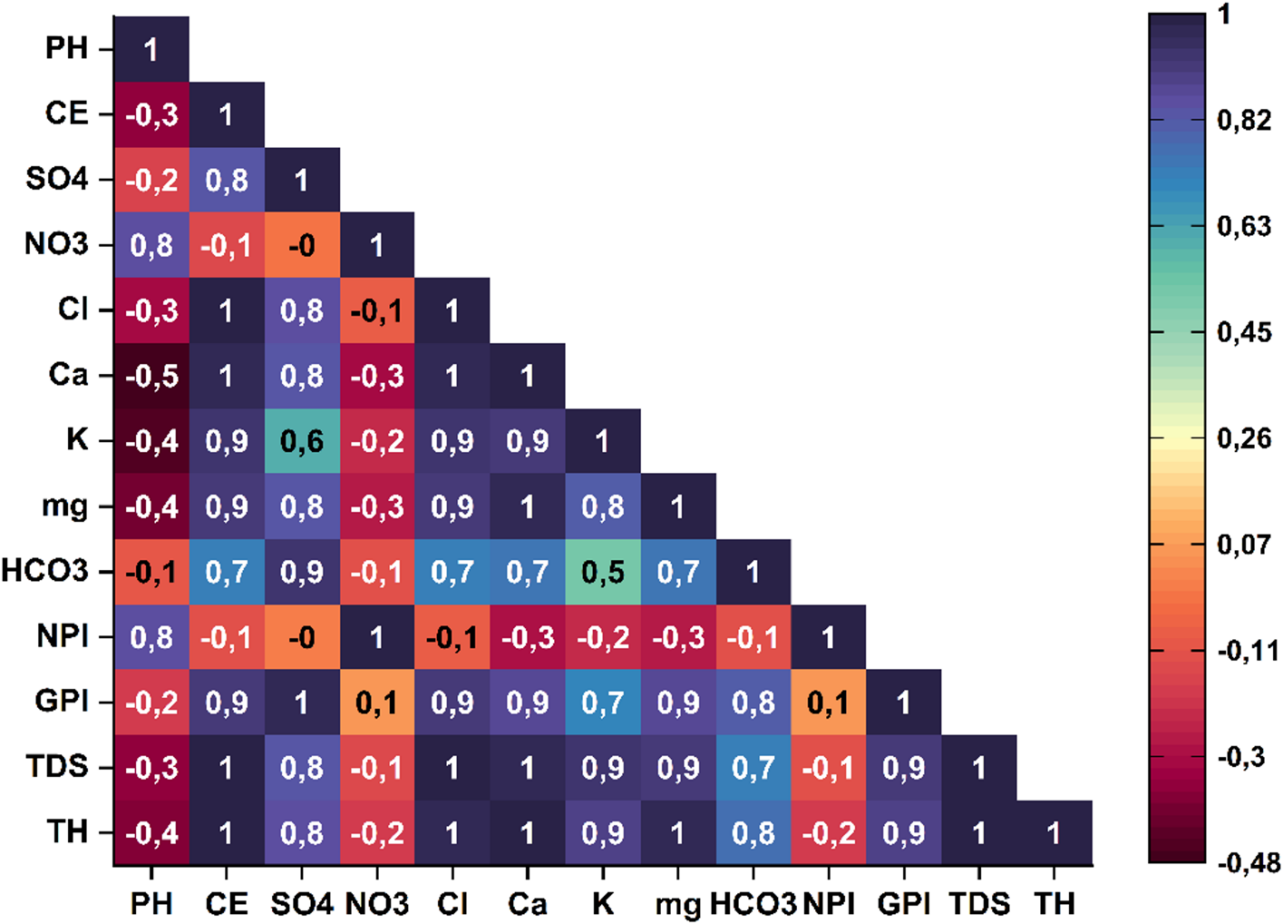
Pearson’s correlation between hydrochemical parameters, GPI and NPI in groundwater of study area.

Correlation analysis serves as a fundamental tool for evaluating the interactions among physicochemical parameters of groundwater and elucidating the processes governing its chemical composition. In the Bouanane basin, Pearson correlation coefficients (Fig. 13) reveal strong positive relationships (*r* > 0.8) among the major dissolved ions, including Cl⁻, SO₄²⁻, Ca²⁺, Mg²⁺, K⁺, TDS, and TH, indicating a common geological origin mainly associated with the dissolution of carbonate and evaporitic formations^35,78,79^. These correlations confirm that the natural mineralization of groundwater primarily results from the dissolution of gypsum (CaSO₄·2 H₂O), halite (NaCl), and carbonate rocks within the aquifer. The strong correlations observed between Cl⁻–Ca²⁺ (*r* = 1), SO₄²⁻–Ca²⁺ (*r* = 0.80), and EC–TDS–TH (*r* = 1) highlight the combined influence of salinity, hardness, and mineralization on electrical conductivity, a key indicator of the total ionic load. The significant correlations among K⁺, Ca²⁺, Mg²⁺, and SO₄²⁻ reflect a dominant lithological control associated with the weathering of clay minerals and feldspars, which progressively release these cations into solution.

The positive correlation between pH and NO₃⁻ (*r* = 0.80) suggests a moderate interaction between natural and anthropogenic processes, likely related to the nitrification of nitrogen compounds or the mineralization of organic matter in agricultural zones. The lack of significant correlations between NO₃⁻ and other major ions indicates that anthropogenic inputs remain limited within the basin. Moreover, the positive correlations between GPI and EC/TDS (*r* = 0.9), the negative correlations between NPI and EC/TDS (*r* = − 0.1), and the strong positive relationship between NPI and NO₃⁻ (*r* = 1), combined with the weak associations between NO₃⁻ and other parameters, suggest that these indices are primarily controlled by overall mineralization and total dissolved salt content^80–82^. These findings emphasize the predominance of geogenic processes in shaping groundwater chemistry, while indicating limited and localized anthropogenic signatures.

### Principal component analysis (PCA)

Principal Component Analysis (PCA) was applied to reduce the dimensionality of the hydrochemical dataset and to identify the dominant factors controlling groundwater composition. All variables were normalized using Z-score standardization to remove scale effects and ensure balanced contributions among parameters. The suitability of the dataset for factor analysis was confirmed by a Kaiser–Meyer–Olkin (KMO) value of 0.82, exceeding the required minimum of 0.5, together with a statistically significant Bartlett’s test (*p* < 0.05), indicating strong inter variable correlations.

The first three principal components account for 96.70% of the total variance, demonstrating the high explanatory power of the PCA model. PC1, which explains 69.7% of the variance, is strongly influenced by SO₄²⁻, HCO₃⁻, Cl⁻, EC, Ca²⁺, and Mg²⁺. This coherent cluster of major ions and electrical conductivity highlights a mineralization–salinity factor governed primarily by geogenic processes, particularly the dissolution of carbonate and evaporitic minerals. The magnitude of this component underscores its central role in defining groundwater chemistry.

The PCA triplot highlights two principal interpretive components. The first component is strongly associated with EC, TDS, Ca²⁺, Mg²⁺, HCO₃⁻, Cl⁻, and SO₄²⁻, indicating that salinity and mineral dissolution processes constitute the dominant hydrochemical controls in the basin. The clustering of these variables suggests a coherent geogenic signature primarily linked to carbonate and evaporite weathering, consistent with the geological framework of the area.

The second component exhibits comparatively weaker loadings related to nitrate and other potential anthropogenic indicators, reflecting a limited but detectable human influence on groundwater composition.

**Fig. 14:**
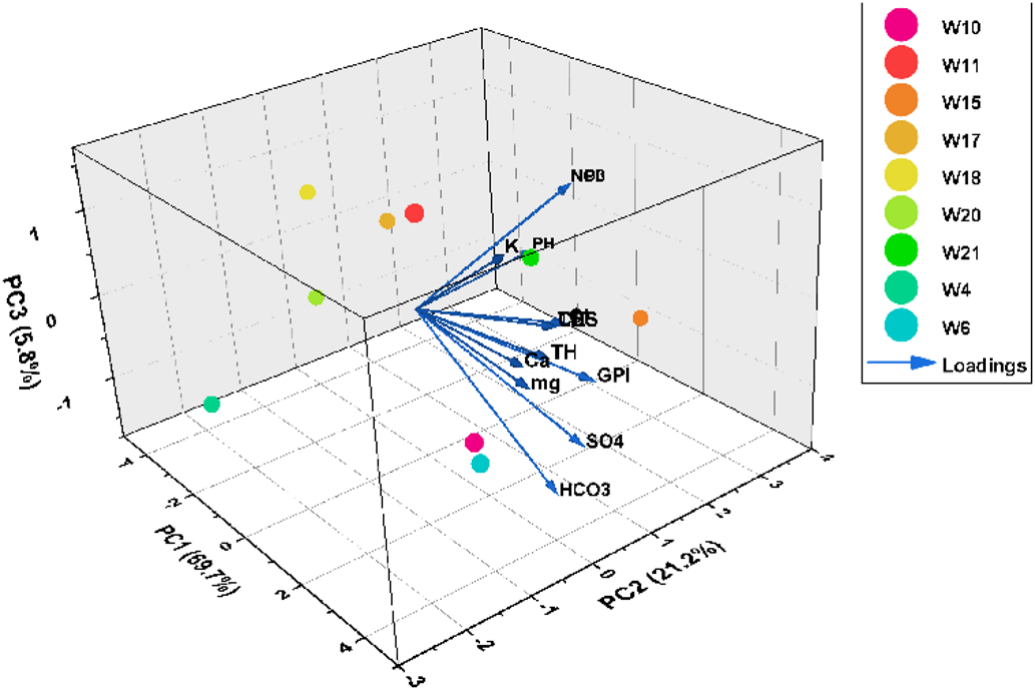
Principal component analysis (PCA) of physicochemical parameters in study area.

Within the scope of the present dataset, PCA therefore supports the interpretation that groundwater salinization and ionic enrichment are predominantly controlled by natural mineralization processes, while anthropogenic contributions remain secondary and spatially localized (Fig. 14).

### Bacteriological quality

Bacteriological analyses performed on all groundwater samples showed a complete absence of coliforms, enterobacteria, and Escherichia coli, indicating broad compliance with Moroccan microbiological standards (MSMAV) and with^1^ drinking water guidelines. This absence of classical fecal indicators suggests that recent fecal contamination is unlikely across most of the aquifer. However, Staphylococcus aureus was detected in 22% of the samples, pointing to localized microbial contamination that may pose health concerns.

Although S. aureus is not traditionally used as a primary fecal contamination indicator, its presence is nevertheless clinically relevant, as it is associated with a range of waterborne illnesses such as gastroenteritis, dysentery, and various opportunistic infections^83,84^. The detected contamination is likely linked to several environmental and infrastructural factors, including infiltration of agricultural wastewater, leakage from poorly sealed or overloaded septic systems, and the absence of adequate sewage collection networks. These conditions facilitate the introduction and persistence of pathogenic bacteria within shallow groundwater systems.

Despite the fact that the chemical quality of groundwater in the Bouanane Basin conforms to WHO standards for major ions and nitrates, the detection of S. aureus highlights the limitations of relying solely on chemical indicators to assess water safety. The findings emphasize the importance of integrating microbiological monitoring into groundwater quality assessments to ensure comprehensive protection of public health. Targeted microbial surveillance, improved wastewater management, and appropriate treatment interventions are therefore essential, particularly in zones where localized contamination has been documented.

### Pollution anthropic sources

**Fig. 15:**
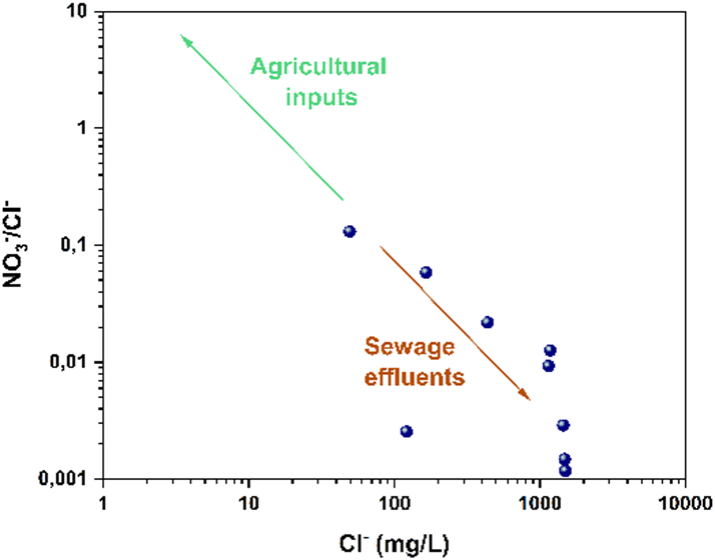
Scatter plots of NO_3_^−^/Cl^−^ versus Cl^−^.

As illustrated in Fig. 15, the sampled groundwater predominantly originated from areas influenced by domestic and municipal inputs, whereas agricultural activities contributed only marginally to nitrate levels. The concentrations of NO₃⁻ associated with agricultural sources remained below drinking water thresholds throughout the study area, confirming the limited role of farming practices in nitrate enrichment. The spatial distribution suggests that residential wastewater infiltration, urban runoff, and inadequate sanitation infrastructure exert a stronger influence on groundwater quality than diffuse agricultural leaching.

These findings are consistent with observations reported elsewhere. Studies conducted in China by^85,86^ similarly demonstrated that groundwater degradation was driven primarily by residential and domestic inputs rather than agricultural sources. Their conclusions highlight a recurring pattern in regions where wastewater management remains insufficient: domestic effluents, even at low to moderate volumes, can impose a greater hydrochemical impact than fertilizer related leaching, particularly when aquifers are shallow or highly permeable.

The correspondence between the present findings and comparable international studies suggests that domestic and municipal discharges represent a discernible anthropogenic influence within the basin. However, this influence appears to be spatially localized and secondary when compared to the dominant lithogenic control exerted by water–rock interactions. While residential effluents may contribute to localized ionic enrichment, particularly in areas lacking adequate wastewater infrastructure, the overall hydrochemical signature of groundwater in the Bouanane Basin remains primarily governed by mineral dissolution processes associated with carbonate and evaporitic formations. Agricultural activities, although present across the landscape, do not currently induce widespread exceedances of drinking water standards, indicating a relatively limited regional impact. Nevertheless, improving wastewater collection and treatment systems remains essential to prevent potential future amplification of anthropogenic pressures on groundwater resources.

.

### Sustainable water management and groundwater resource protection

The sustainability of groundwater resources in the Bouanane Basin requires an integrated management approach that considers both chemical and microbiological water quality as well as the anthropogenic pressures impacting the aquifer. Although physicochemical parameters comply with WHO guidelines, the occasional detection of *Staphylococcus aureus* and the influence of domestic and municipal inputs reveal localized vulnerabilities. Strengthening sanitation infrastructure, improving wastewater collection and treatment, and establishing protection zones around wells are essential measures to mitigate contamination risks.

Moreover, continuous and systematic monitoring of hydrochemical and microbiological parameters is crucial for the early detection of emerging risks and for guiding corrective interventions. While agricultural activities represent a secondary influence, the adoption of sustainable practices such as optimized fertilizer application, soil conservation, and improved irrigation efficiency remains important to minimize additional stress on the aquifer. Finally, long term groundwater sustainability depends on integrated governance involving local authorities, water managers, and communities, combining effective infrastructure, scientific monitoring, and community engagement to ensure the resilience and quality of groundwater resources.

## Conclusion

In semi-arid and arid regions, groundwater pollution represents a strategic challenge due to fragile natural recharge rates, increasing water demand, intensified evaporation effects, and the limited capacity of hydrological systems to dilute contaminants. Within this context, this comprehensive assessment of groundwater quality in the Bouanane Basin demonstrates that geogenic processes, particularly the dissolution of carbonate and evaporitic rocks, are the primary drivers of elevated salinity and hardness. Although most physicochemical parameters remain within or close to WHO (2017b) standards, several downstream wells exhibit high electrical conductivity, total dissolved solids, and hardness, reflecting intensified mineralization along the groundwater flow path. Very low nitrate levels and low NPI values indicate limited anthropogenic influence, supporting the aquifer’s resilience to agricultural and domestic pressures. Conversely, the intermittent detection of Staphylococcus aureus reveals localized contamination hotspots linked to deficiencies in wastewater services, while the GPI classifies four wells as “very highly” polluted, highlighting spatial variability associated with geological and hydrodynamic features of the basin.

The findings based on nine sampling points and reflect conditions during the April 2024 campaign, confirm that the combined use of GPI, NPI, and health risk assessment offers a robust and comprehensive diagnostic framework capable of accurately evaluating groundwater quality and associated human health risks. As the first in depth hydro-environmental assessment conducted in southeastern Morocco, this study provides a solid scientific basis to support sustainable groundwater governance. Preserving this vital resource under climate variability and increasing anthropogenic pressures requires strengthening long-term monitoring programs, improving wastewater and sanitation infrastructure, and promoting responsible agricultural input use to reinforce system resilience.

In conclusion, this study recommends directing future scientific efforts toward integrating environmental isotope techniques and geochemical modeling to more precisely identify mineralization sources, applying remote sensing and climate modeling to estimate groundwater recharge under changing climatic conditions, and exploring the potential of managed aquifer recharge to enhance long term water security. Enhanced collaboration between research institutions, policymakers, and local stakeholders will be essential for developing a more integrated and resilient groundwater management strategy that ensures resource sustainability in arid and semi-arid regions.

## Data Availability

All data generated or analyzed during this study are included in this published article.
